# Ambulatory hypertension diagnosed by 24-h mean ambulatory versus day and night ambulatory blood pressure thresholds in children: a cross-sectional study

**DOI:** 10.1186/s40885-022-00217-2

**Published:** 2022-11-15

**Authors:** Ajay P. Sharma, Luis Altamirano-Diaz, Mohamed Mohamed Ali, Katryna Stronks, Amrit Kirpalani, Guido Filler, Kambiz Norozi

**Affiliations:** 1 Division of Nephrology, Department of Paediatrics, University of Western Ontario, London, ON Canada; 2 London Health Sciences Centre, London, ON Canada; 3 Division of Cardiology, Department of Paediatrics, University of Western Ontario, London, ON Canada; 4 University of Western Ontario, London, ON Canada; 5 Department of Paediatric Cardiology and Intensive Care, Medical School Hannover, Hannover, Germany

**Keywords:** Pediatrics, Blood Pressure, Hypertension, White coat hypertension, Masked hypertension, Ambulatory Blood Pressure Monitoring

## Abstract

**Background:**

The agreement between the commonly used ambulatory blood pressure (ABP) thresholds to diagnose ambulatory hypertension in children (patient’s 24-h mean ABP classified by 24-h 95th ABP percentile threshold, American Heart Association [AHA] threshold, or patient’s day and night mean ABP classified by day-night 95th ABP percentile thresholds) is not known. We evaluated the agreement among 24-h ABP threshold, AHA threshold, and day-night ABP thresholds to diagnose ambulatory hypertension, white coat hypertension (WCH) and masked hypertension (MH).

**Methods:**

In a cross-sectional study design, we analyzed ABP recordings from 450 participants with suspected hypertension from a tertiary care outpatient hypertension clinic. The American Academy of Pediatrics thresholds were used to diagnose office hypertension.

**Results:**

The 24-h ABP threshold and day-night ABP thresholds classified 19% ABP (95% confidence interval [CI], 0.15–0.23) differently into ambulatory normotension/hypertension (kappa [κ], 0.58; 95% CI, 0.51–0.66). Ambulatory hypertension diagnosed by 24-h ABP threshold in 27% participants (95% CI, 0.22–0.32) was significantly lower than that by day-night ABP thresholds in 44% participants (95% CI, 0.37–0.50; *P* < 0.001). The AHA threshold had a stronger agreement with 24-h ABP threshold than with day-night ABP thresholds for classifying ABP into ambulatory normotension/hypertension (k 0.94, 95% CI 0.91–0.98 vs. k 0.59, 95% CI 0.52–0.66). The diagnosis of ambulatory hypertension by the AHA threshold (26%; 95% CI, 0.21–0.31) was closer to that by 24-h ABP threshold (27%, *P* = 0.73) than by day-night ABP thresholds (44%, *P* < 0.001). Similar agreement pattern persisted among these ABP thresholds for diagnosing WCH and MH.

**Conclusions:**

The 24-h ABP threshold classifies a lower proportion of ABP as ambulatory hypertension than day-night ABP thresholds. The AHA threshold exhibits a stronger agreement with 24-h ABP than with day-night ABP thresholds for diagnosing ambulatory hypertension, WCH and MH. Our findings are relevant for a consistent interpretation of hypertension by these ABP thresholds in clinical practice.

## Background

The American Heart Association (AHA) and European Society of Hypertension recommend the use of ambulatory blood pressure monitoring (ABPM) in clinical practice to diagnose ambulatory hypertension [1, 2]. Ambulatory normotension or hypertension status helps to classify office blood pressure (OBP) into white coat hypertension (WCH) and masked hypertension (MH) [1–5]. In children, patient’s 24-h/day/night mean ambulatory blood pressure (ABP) values are interpreted by the age-, sex-, and height-specific 24-h/day/night 95th systolic/diastolic ABP percentiles [6, 7].

Based on the assumption that 24-h ABP represents both day and night ABP, ambulatory hypertension in children is commonly diagnosed by either patient’s 24-h mean ABP exceeding the 24-h 95th ABP percentile (24-h ABP threshold) or patient’s mean day/night ABP exceeding the day/night 95th ABP percentiles, respectively (day-night ABP thresholds) [8–13]. The AHA guidelines have recommended a modified version of 24-h 95th ABP percentile (24-h 95th ABP percentile along with 24-h ABP load ≥ 25%) [1, 3]. However, the agreement between 24-h ABP, day-night ABP, and AHA thresholds to diagnose ambulatory hypertension is not known.

Based on the observations that an individual can have a disconnect in the diagnosis of day and night ambulatory hypertension based on the corresponding day/night 95th ABP percentiles [14–18], we hypothesized that 24-h ABP threshold may diagnose ambulatory hypertension differently than day-night ABP thresholds. Therefore, our primary objective was to compare the diagnosis of ambulatory hypertension by the 24-h ABP and day-night ABP thresholds, as well to evaluate their relative agreement with the AHA threshold to diagnose ambulatory hypertension. Our secondary objective was to assess the agreement among these ABP thresholds to diagnose WCH and MH.

## Methods

This was a single center, retrospective cross-sectional study performed after an approval by the Research Ethics Board of the University of Western Ontario (No. 16143E and 116,082). The study involved the retrospective review of existing clinical data and was therefore exempted from the need for an individual informed consent. The records of children who underwent ABPM at a tertiary care outpatient hypertension clinic (London, ON, Canada) were collected. The data was collected between January 2003 and December 2008 (*n* = 159) as a part of previous studies and recently between January 2018 and September 2020 (*n* = 291) [19–23]. The participants were referred to our outpatient clinic at London Health Sciences Center because of suspected hypertension based on OBP assessments by the primary health care providers. During both the study periods, there was a uniformity in the protocol regarding offering ABPM to all patients older than 5 years and for evaluating secondary hypertension (the fourth report guidelines [24] during the first study period and similar recommendations by the American Academy of Pediatrics [AAP] guidelines during the second period [3]). The participants with an inadequate ABPM or missing OBP recordings were excluded. Anthropometric measurements including age, weight, and height were collected. Body mass index (BMI) was calculated as the weight (kg)/height (cm)^2^. BMI percentiles were calculated based on the Centers for Disease Control and Prevention reference intervals (overweight, 85th–95th percentiles; obese, > 95th percentile) [25].

### Office blood pressure measurement

We performed OBP measurements by the methodology recommended by the fourth report and the AAP guidelines [3, 24]. In brief, a trained nurse measured OBP in a quiet state with child seated for 3 to 5 min, back supported, using an appropriate-sized cuff selected according to child’s upper right arm. OBP was initially measured by a calibrated oscillometric device (V 100; Dinamap, Tampa, FL, USA) [26]. If oscillometric OBP measurements remain elevated (≥ 90th percentile), auscultatory OBP measurements were performed using a calibrated aneroid sphygmomanometer [3, 24]. An average of last two auscultatory OBP measurements was used to diagnose office hypertension [3, 24].

### Ambulatory blood pressure measurement

A 24-h ABPM was performed with an oscillometric ambulatory BP monitors (model 90,207; Spacelabs Inc., Redmond, WA, USA) [6, 27]. A trained nurse chose an appropriate-sized cuff and conducted the ABPM as per the fourth report and AAP guidelines [3, 24]. The cuff was placed on the nondominant arm, with ABP recordings planned for every 20 min during day and 30 min during night [1, 3]. The participants were instructed to continue with their regular daily activity, to avoid strenuous exercise, and to maintain a wake-sleep log for defining day and night ABP [28]. The adequacy of ABP recordings was established based on minimum one reading per hour during day and nighttime, and more than 40 readings in 24-h [3]. The 24-h, day and night systolic and diastolic ABP on each patient were analyzed by the respective 24-h/day/night 95th ABP percentile references, recommended by Wühl et al. [6] and endorsed by the AAP guidelines [3].

### Outcomes

Our primary outcome was to compare ambulatory hypertension by the 24-h, day-night, and AHA ABP thresholds. Our secondary outcome was to evaluate WCH and MH by these ABP thresholds.

### Definitions

- Office hypertension as per the AAP guidelines: patient’s systolic or diastolic OBP ≥ age-, sex-, and height-specific 95th systolic/diastolic OBP percentiles or OBP ≥ 130/80 for patients ≥ 13 years [3].
- Ambulatory hypertension as per 24-h ABP threshold: patient’s 24-h mean systolic or diastolic ABP ≥ 24-h age-, sex-, and height-specific 95th systolic/diastolic ABP percentiles [6].
- Ambulatory hypertension as per day−night ABP thresholds: patient’s day or night mean systolic or diastolic ABP ≥ corresponding day/night age−, sex−, and height−specific 95th systolic/diastolic ABP percentiles [6].
- Ambulatory hypertension as per the AHA threshold: patient’s 24-h mean systolic or diastolic ABP ≥ 24-h age-, sex-, and height-specific 95th systolic/diastolic ABP percentiles and 24-h systolic or diastolic ABP load ≥ 25% [1, 3]. A 24-h systolic/diastolic ABP load was calculated as the percentage of ABP measurements higher than the 24-h mean 95th systolic/diastolic ABP percentiles [6].

### Statistical methods

Normally distributed continuous variables were reported as mean (standard deviation, SD), otherwise as median (interquartile range, IQR). Categorical variables were reported as frequency and percentage. Continuous variables were compared with the parametric unpaired t-test or the nonparametric Mann–Whitney U-test, as appropriate. Categorical variables were compared with chi-square test. Systolic and diastolic OBP z-scores and percentiles were calculated based on the methodology recommended by the AAP guidelines [3, 29]. The 24-h, day and night systolic/diastolic ABP z-score, and 95th ABP percentiles were calculated using Box-Cox transformations with age-, and sex-specific estimates of the distribution median, coefficient of variation, and degree of skewness recommended by Wuhl et al. [6]. The agreement between the ABP thresholds was calculated by the proportion of ABP classified similarly (accuracy) into ambulatory normotension/hypertension and by using the kappa (κ) statistics [30]. Adolescents were defined as those with age ≥ 13 years, as recommended by the AAP guidelines [3]. Accuracy and kappa statistics were calculated on MedCalc ver. 18.11 (MedCalc Software, Ostend, Belgium). All other statistical analysis was performed IBM SPSS ver. 25.0 (IBM Corp., Armonk, NY, USA).

## Results

### Patient characteristics

In the initial screening, 544 participants who had ABPM studies during the recruitment period met the inclusion criteria. Ninety-four participants were excluded for the following reasons: 53 had less than two OBP recordings and 41 had an inadequate ABPM. Four hundred and fifty eligible participants aged 5 to 18 years with complete ABPM and OBP recordings were included in this analysis. The study sample included 59% adolescents ≥ 13 years, 59% males, and 55% overweight/obese participants. Each participant was included with a single ABPM recording in the analysis. The 24-h ABP threshold diagnosed ambulatory hypertension in 122 (27%; 95% confidence interval [CI], 0.22–0.32) and day-night ABP thresholds in 197 participants (44%; 95% CI, 0.37–0.50). Ambulatory hypertension by 24-h ABP threshold and day-night ABP thresholds did not differ in age, sex, BMI z-score, overweight/obese participants, office hypertension, primary hypertension, and blood pressure medication intake; however, ambulatory hypertension by day-night ABP thresholds had more adolescents and ambulatory hypertension by 24-h ABP threshold had higher systolic and diastolic ABP z-scores and ABP loads (Table 1) [3, 6]. As the data was included from two different time periods, we compared the patient characteristics over the two periods, which did not show a significant difference in first versus second period in age (mean ± SD, 12.98 ± 3.95 years vs. 13.06 ± 3.37 years; *P* = 0.83), males (60% vs. 59%; *P* = 0.11), BMI z-score (median [IQR], 1.10 [0.26–2.02] vs. 1.23 [0.12–1.99]; *P* = 0.70) and overweight/obese (54% vs. 56%; *P* = 0.10) distribution.

**Table 1:** Patient characteristics

Characteristic	Entire group (*n* = 450)	(A) Ambulatory hypertension by 24-h mean ABP threshold (*n* = 122)	(B) Ambulatory hypertension by day-night ABP thresholds (*n* = 197)	*P*-value (A vs. B)
Age (yr)	13.03 ± 3.58	12.68 ± 3.73	13.27 ± 3.54	0.15
≥ 13	267 (59)	59 (48)	120 (61)	0.02
Male sex	267 (59)	75 (61)	114 (58)	0.59
Overweight/obese	248 (55)	70 (57)	116 (59)	0.72
BMI z-score	1.21 (0.16 to 2.00)	1.46 (0.38 to 2.18)	1.32 (0.31 to 2.05)	0.50
Office hypertension	57 (36)	93 (76)	131 (66)	0.06
OBP systolic z score	1.68 (0.33 to 2.30)	2.28 (1.51 to 3.00)	2.09 (1.25 to 2.78)	0.22
OBP diastolic z-score	0.60 (–0.05 to 1.41)	1.04 (0.37 to 1.64)	0.99 (0.33 to 1.53)	0.62
Primary hypertension	352 (78)	109 (89)	174 (88)	0.78
Secondary hypertension	98 (22)	13 (11)	23 (12)	0.78
No BP medication	322 (72)	102 (84)	164 (83)	0.81
BP medication	128 (28)	20 (16)	33 (17)	0.81
ABP systolic z-score	0.32 (–0.60 to 1.18)	1.94 (1.27 to 2.66)	1.29 (0.51 to 2.16)	< 0.001
ABP systolic load (%)	21.42 (7.14 to 44.44)	60.85 (41.40 to 80.00)	42.31 (22.47 to 65.63)	< 0.001
ABP diastolic z-score	0.13 (–0.69 to 1.18)	1.78 (1.07 to 2.65)	1.23 (0.40 to 2.03)	< 0.001
ABP diastolic load (%)	15.38 (6.67 to 34.20)	41.55 (28.20 to 60.47)	34.62 (16.95 to 51.06)	< 0.001

Data are presented as mean ± standard deviation, number (%), or median (interquartile range). Office hypertension was diagnosed by the American Academy of Pediatrics thresholds [3]. 24-h mean ABP threshold: 24-h mean ABP ≥ 24-h 95th ABP percentile according to the ABPM references [6]; Day-night ABP thresholds: mean day ABP ≥ day 95th ABP percentile [6] or mean night ABP ≥ night 95th ABP percentile [6]

*ABP* ambulatory blood pressure, *BMI* body mass index, *OBP* office blood pressure, *BP* blood pressure

### Diagnosis of ambulatory hypertension, WCH, and MH by 24-h ABP vs. day-night ABP thresholds

**Fig. 1:**
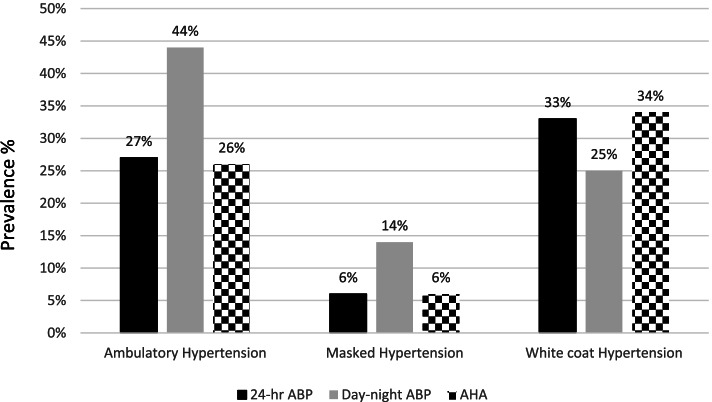
Ambulatory hypertension (AH), masked hypertension (MH), and white coat hypertension (WCH) as per the 24-h ambulatory blood pressure (ABP), day-night ABP and American Heart Association (AHA) thresholds. The 24-h ABP threshold diagnosed fewer AH (27% vs. 44%; *P* < 0.001), MH (6% vs. 14%; *P* < 0.001), and more WCH (33% vs. 25%; *P* < 0.001) than day-night ABP thresholds. The 24-h ABP and AHA thresholds diagnosed AH (27% vs. 26%; *P* = 0.73), MH (6% vs. 6%; P > 0.999), and WCH (33% vs. 34%; *P* = 0.75) quite similarly, whereas the day-night ABP and AHA thresholds diagnosed AH (44% vs. 26%; *P* < 0.001), MH (14% vs. 6%; *P* < 0.001), and WCH (25% vs. 34%; *P* < 0.001) differently

The 24-h ABP threshold and day-night ABP thresholds demonstrated only a moderate agreement (κ, 0.58; 95% CI, 0.51–0.66) by classifying 81% ABP (95% CI, 0.72–0.89) similarly into ambulatory normotension/hypertension. As a result, 24-h ABP threshold diagnosed fewer ambulatory hypertension (27%; 95% CI, 0.22–0.32) than day-night ABP thresholds (44%; 95% CI, 0.37–0.50; *P* < 0.001) (Table 2, Fig. 1) [6]. Fewer ambulatory hypertension by 24-h ABP threshold translated into more WCH (33%, 95% CI 0.28–0.39 vs. 25%, 95% CI 0.20–0.30; *P* < 0.001) and fewer MH (6%, 95% CI 0.04–0.09 vs. 14%, 95% CI 0.11–0.18; *P* < 0.001) by 24-h ABP than day-night ABP thresholds (Table 2, Fig. 1) [6]. The pattern of ambulatory hypertension, WCH, and MH diagnosis by the 24-h ABP and day-night ABP thresholds remained consistent across the age, sex, BMI, primary hypertension, and no BP medication intake (Table 2) [6].

**Table 2:** Diagnosis of ambulatory hypertension, white coat hypertension, and masked hypertension based on 24-h mean ABP and day-night ABP thresholds

Variable	24-h mean ABP threshold (%; 95% CI)	Day-night ABP thresholds (%; 95% CI)	Difference (%; 95% CI)
Ambulatory hypertension			
Entire group (*n* = 450)	27 (0.22 to 0.32)	44 (0.37 to 0.50)	17 (10.8 to 23)^*^
Age ≥ 13 yrs. (*n* = 267)	22 (0.16 to 0.28)	45 (0.37 to 0.53)	23 (15.1 to 30.5)^*^
Male sex (*n* = 267)	28 (0.22 to 0.35)	43 (0.35 to 0.51)	15 (6.9 to 22.8)^*^
Overweight/obese (*n* = 248)	28 (0.22 to 0.35)	47 (0.38 to 0.56)	19 (10.5 to 27.1)^*^
Primary hypertension (*n* = 352)	31 (0.25 to 0.37)	49 (0.42 to 0.57)	18 (10.8 to 24.9)^*^
Secondary hypertension (*n* = 98)	13 (0.07 to 0.22)	23 (0.14 to 0.35)	10 (0.8 to 20.7)
No BP medication (*n* = 322)	32 (0.25 to 0.38)	51 (0.43 to 0.59)	19 (11.4 to 26.3)^*^
On BP medication (*n* = 128)	16 (0.09 to 0.24)	26 (0.17 to 0.36)	10 (0.01 to 19.8)
White coat hypertension			
Entire group (*n* = 450)	34 (0.28 to 0.39)	25 (0.20 to 0.30)	9 (2.1 to 13.9)^*^
Age ≥ 13 yr (*n* = 267)	35 (0.28 to 0.43)	23 (0.18 to 0.30)	12.0 (4.3 to 19.5)^*^
Male sex (*n* = 267)	33 (0.27 to 0.41)	26 (0.20 to 0.33)	70 (–0.7 to 14.6)
Overweight/obese (*n* = 248)	37 (0.30 to 0.46)	25 (0.19 to 0.32)	12 (3.9 to 19.9)^*^
Primary hypertension (*n* = 352)	34 (0.29 to 0.41)	25 (0.20 to 0.31)	9 (2.3 to 15.6)^*^
Secondary hypertension (*n* = 98)	28 (0.18 to 0.41)	23 (0.14 to 0.35)	5 (–7.2 to 17)
No BP medication (*n* = 322)	36 (0.29 to 0.43)	25 (0.20 to 0.31)	11 (3.9 to 18)^*^
On BP medication (*n* = 128)	27 (0.19 to 0.38)	23 (0.15 to 0.33)	4 (–6.6 to 14.5)
Masked hypertension			
Entire group (*n* = 450)	6 (0.04 to 0.09)	14 (0.11 to 0.18)	8 (4.1 to 12)^*^
Age ≥ 13 yr (*n* = 267)	6 (0.03 to 0.10)	17 (0.12 to 0.23)	11 (5.6 to 16.5)^*^
Male sex (*n* = 267)	6 (0.03 to 0.10)	14 (0.10 to 0.19)	8 (2.9 to 13.2^)*^
Overweight/obese (*n* = 248)	6 (0.03 to 0.10)	12 (0.08 to 0.17)	8 (2.9 to 13.2)^*^
Primary hypertension (*n* = 352)	6 (0.04 to 0.09)	15 (0.11 to 0.20)	9 (4.5 to 13.6)^*^
Secondary hypertension (*n* = 98)	6 (0.02 to 0.13)	11 (0.05 to 0.20)	5 (–3.1 to 13.4)
No BP medication (*n* = 322)	8 (0.05 to 0.12)	17 (0.13 to 0.22)	9 (3.9 to 14.1)^*^
On BP medication (*n* = 128)	1 (0.00 to 0.05)	7 (0.03 to 0.14)	6 (1.1 to 11.8)^*^

Office hypertension was diagnosed by the American Academy of Pediatrics thresholds [3]. 24-h mean ABP threshold: 24-h mean ABP ≥ 24-h 95th ABP percentile according to the ABPM references [6]; Day-night ABP thresholds: mean day ABP ≥ day 95th ABP percentile [6] or mean night ABP ≥ night 95th ABP percentile [6]

*ABP* ambulatory blood pressure, *CI* confidence interval, *BP* blood pressure, *ABPM* ambulatory blood pressure monitoring

^*^P < 0.05

### Agreement between 24-ABP and day ABP vs. night ABP thresholds

Despite the fact that 24-h ABP threshold reflects the mean of day and night ABP thresholds, only a moderate agreement between the 24-h and day-night ABP thresholds led us to look at the individual association between the 24-h ABP threshold and day ABP vs. night ABP threshold. We found that 24-h ABP threshold had a stronger agreement with day ABP (91%; 95% CI, 0.82–1.06) than night ABP (78%; 95% CI, 0.70–0.87; *P* < 0.001) to classify ABP into ambulatory normotension/hypertension (Table 3) [6]. Therefore, the diagnosis of ambulatory hypertension was closer between 24-h ABP and day ABP threshold (difference, 3.70%; 95% CI, 1.97%–9.34%; *P* = 0.20) than between 24-h ABP and night ABP threshold (difference, 11%; 95% CI, 4.9%–17%; *P* < 0.001). The agreement pattern between 24-h ABP and day ABP versus night ABP threshold remained consistent across the age, sex, BMI, primary hypertension, and no BP medication intake (Table 3) [6].

**Table 3:** Agreement between 24-h mean ABP and day versus night ABP for diagnosing ambulatory normotension/hypertension

Variable	Accuracy (%)^a)^ (95th CI)	Kappa (95% CI)
Day ABP threshold		
Entire group (*n* = 450)	91 (0.82–1.06)	0.77 (0.70–0.83)
Age ≥ 13 yr (*n* = 267)	91 (0.80–1.03)	0.73 (0.63–0.83)
Male sex (*n* = 267)	92 (0.81–1.05)	0.82 (0.74–0.89)
Overweight/obese (*n* = 248)	91 (0.79–1.03)	0.77 (0.69–0.86)
Primary hypertension (*n* = 352)	90 (0.80–1.01)	0.77 (0.69–0.84)
Secondary hypertension (*n* = 98)	93 (0.75–1.15)	0.71 (0.50–0.93)
No BP medication (*n* = 322)	90 (0.79–1.01)	0.76 (0.68–0.84)
On BP medication (*n* = 128)	94 (0.78–1.12)	0.76 (0.60–0.93)
Night ABP threshold		
Entire group (*n* = 450)	78 (0.70–0.87)	0.52 (0.44–0.60)
Age ≥ 13 yr (*n* = 267)	73 (0.63–0.84)	0.39(0.29–0.50)
Male sex (*n* = 267)	78 (0.68–0.90)	0.57(0.47–0.68)
Overweight/obese (*n* = 248)	79 (0.69–0.91)	0.55(0.45–0.66)
Primary hypertension (*n* = 352)	76 (0.67–0.85)	0.49 (0.40–0.58)
Secondary hypertension (*n* = 98)	88 (0.71–1.09)	0.60 (0.39–0.81)
No BP medication (*n* = 322)	75 (0.66–0.85)	0.48 (0.39–0.58)
On BP medication (*n* = 128)	87 (0.72–1.05)	0.59 (0.41–0.76)

24-h mean ABP threshold: 24-h ABP ≥ 24-h 95th ABP percentile according to the ABPM references [6]; Day ABP threshold: mean day ABP ≥ day 95th ABP percentile [6]; Night ABP threshold: mean night ABP ≥ night 95th ABP percentile [6]

*ABP* ambulatory blood pressure, *CI* confidence interval, *BP* blood pressure, *ABPM* ambulatory blood pressure monitoring

^a)^The proportion of ABP classified similarly by both the ABP thresholds into ambulatory normotension/hypertension

### Agreement between the AHA threshold and 24-h ABP vs. day-night ABP thresholds

The AHA threshold demonstrated a strong agreement with 24-h ABP threshold (κ, 0.94; 95% CI, 0.91–0.98), and the two thresholds classified 98% ABP (95% CI, 0.89–1.07) similarly into ambulatory normotension/hypertension (Table 4) [3, 6]. In contrast, the AHA threshold showed only a moderate agreement with day-night ABP thresholds (κ, 0.59; 95% CI, 0.52–0.66), with a relatively lower proportion of ABP classified similarly by the two ABP thresholds (80%; 95% CI, 0.72–0.89) (Table 4) [3, 6]. The stronger agreement between the AHA and 24-h ABP threshold than with the day-night ABP thresholds remained consistent in sub-group analysis on adolescent, male, overweight-obese, primary hypertension, and no BP medication intake (Table 4) [3, 6].

**Table 4:** Agreement between the AHA threshold and the 24-h mean ABP threshold versus day-night ABP thresholds for diagnosing ambulatory normotension/hypertension

Variable	AHA and 24-h mean ABP threshold		AHA and Day-night ABP thresholds	
	Accuracy (%)^a)^ (95th CI)	Kappa (95% CI)	Accuracy (%)^a)^ (95th CI)	Kappa (95% CI)
Entire group (*n* = 450)	98 (0.89–1.07)	0.94 (0.91–0.98)	80 (0.72–0.89)	0.59 (0.52–0.66)
Age ≥ 13 yr (*n* = 267)	98 (0.86–1.10)	0.94(0.89–0.99)	76 (0.65–0.87)	0.49(0.39–0.58)
Male sex (*n* = 267)	97 (0.85–1.09)	0.93(0.88–0.98)	83 (0.72–0.95)	0.64(0.55–0.73)
Overweight/obese (*n* = 248)	97 (0.85–1.10)	0.93 (0.89–0.98)	79 (0.69–0.91)	0.58(0.49–0.67)
Primary hypertension (*n* = 352)	97 (0.87–1.08)	0.93 (0.90–0.97)	78 (0.69–0.88)	0.56 (0.48–0.64)
Secondary hypertension (*n* = 98)	98 (0.81–1.21)	1.00 (1.00–1.00)	89 (0.72–1.10)	0.66 (0.48–0.85)
No BP medication (*n* = 322)	97 (0.86–1.08)	0.93 (0.89–0.97)	77 (0.69–0.87)	0.54 (0.46–0.63)
On BP medication (*n* = 128)	100 (0.83–1.18)	1.00 (1.00–1.00)	89 (0.74–1.07)	0.69 (0.54–0.84)

24-h mean ABP threshold: 24-h mean ABP ≥ 24-h 95th ABP percentile according to the ABPM references [6]. Day-night ABP thresholds: day ABP ≥ 95th day ABP percentile [6] or mean night ABP ≥ 95th night ABP percentile [6]. AHA threshold: 24-h systolic or diastolic ABP ≥ 95th ABP percentile and 24-h systolic or diastolic ABP load ≥ 25% [3]. 24-h-ABP load: proportion of systolic or diastolic ABP readings higher than 24-h mean systolic or diastolic 95th ABP percentile

*AHA* American Heart Association, *ABP* ambulatory blood pressure, *CI* confidence interval, *BP* blood pressure, *ABPM* ambulatory blood pressure monitoring

^a)^The proportion of ABP classified similarly by both the ABP thresholds into ambulatory normotension/hypertension

The stronger agreement between the AHA and 24-h ABP thresholds translated into AHA threshold diagnosing ambulatory hypertension closer to 24-h ABP threshold (26%; 95% CI, 0.21–0.31 vs. 27%; 95% CI, 0.22–0.32; *P* = 0.73) than day-night ABP thresholds (44%; 95% CI, 0.37–0.50; *P* < 0.001) (Fig. 1). Similarly, the diagnosis of WCH (34%; 95% CI, 0.29–0.40) and MH (6%; 95% CI, 0.04–0.09) by the AHA threshold was not significantly different than WCH (33%; 95% CI, 0.28–0.39; *P* = 0.75) and MH (6%; 95% CI, 0.04–0.09; P > 0.999 by the 24-h ABP threshold (Fig. 1). However, the diagnosis of WCH (25%; 95% CI, 0.20–0.30) and MH (14%; 95% CI, 0.11–0.18) by the day-night ABP thresholds was significantly different than WCH (difference, 9%; 95% CI, 3%–14.9%; *P* < 0.001) and MH (difference, 8%; 95% CI, 4.1%–12%; *P* < 0.001) by the AHA threshold (Fig. 1).

## Discussion

The choice of an ABP threshold can influence the diagnosis of ambulatory hypertension, consequently that of WCH/MH [8]. In clinical practice, 24-h ABP, day-night ABP, and AHA thresholds are commonly used to diagnose ambulatory hypertension [8–13]. We found that 24-h ABP threshold diagnosed significantly fewer ambulatory hypertension than day-night ABP thresholds, which reflected in the difference in the diagnosis of WCH and MH by these ABP thresholds. In addition, the AHA threshold diagnosed ambulatory hypertension, WCH, and MH closer to 24-h ABP threshold than day-night ABP thresholds.

Given the paucity of literature on the agreement between these commonly used ABP thresholds, our findings become novel and important for a consistent interpretation of ambulatory hypertension by these ABP thresholds in clinical practice. We found that 24-h ABP and day-night ABP thresholds classified ABP similarly into ambulatory normotension/hypertension in 81% participants, which can be explained by the fact that patient’s 24-h ABP is expected to represent an average of the day and night ABP measurements. However, the noteworthy observation from our analysis was that 24-h ABP threshold diagnosed 17% fewer ambulatory hypertension than the day-night ABP thresholds (27% vs. 44%). As a result, 24-h ABP threshold diagnosed more WCH and fewer MH than the day-night ABP thresholds, which remained consistent across subgroups based on age, sex, BMI, primary hypertension, and no BP medication intake. Our finding on the discrepancy in the diagnosis of ambulatory hypertension by the 24-h and day-night ABP thresholds remains consistent with the adult literature that demonstrated a difference in ambulatory hypertension by the 24-h ABP threshold versus 24-h ABP threshold combined with day and night ABP thresholds [31, 32]. Based on these observations, it can be concluded that 24-h ABP and day-night ABP thresholds diagnose ambulatory hypertension differently, which can impact the determination of WCH and MH by these thresholds.

The difference in the diagnosis of ambulatory hypertension by 24-h ABP versus day-night ABP thresholds can be explained by a relatively stronger agreement between 24-h ABP and day ABP than between 24-h ABP and night ABP threshold. We found that 24-h ABP and day ABP thresholds classified 91% ABP similarly into ambulatory normotension/hypertension, which was higher than 78% ABP classified similarly by the 24-h and night ABP thresholds. It is tempting to speculate that the stronger agreement between the 24-h ABP and day ABP threshold than with the night ABP threshold results from a relatively higher representation of day ABP on 24-h ABPM, which can be explained by the standard recommendations of measuring day ABP more frequently, every 20 to 30 min, than more spread out night ABP recordings every 30 to 40 min [1, 3, 24]*.*

Given the fact that the AHA guidelines have recommended a different ABP threshold than 24-h and day-night ABP thresholds to diagnose ambulatory hypertension, we evaluated the agreement between the AHA threshold and 24-h ABP/day-night ABP thresholds. We found that the AHA threshold had a stronger agreement with 24-h ABP threshold than with day-night ABP thresholds. The AHA and 24-h ABP thresholds classified 90% ABP similarly into ambulatory normotension/hypertension, which was higher than 80% ABP classified similarly by the AHA and day-night ABP thresholds. The stronger agreement between the AHA and 24-h ABP thresholds persisted regardless of age, sex, BMI, primary hypertension, or no antihypertensive medication intake. The stronger agreement between the AHA and 24-h ABP than day-night ABP thresholds can be explained by the fact that the AHA threshold is based on 24-h mean 95th ABP percentile along with ABP load ≥ 25% estimated by 24-h mean 95th ABP percentile. As a result, it can be concluded that day-night ABP threshold and AHA threshold cannot be interchangeably used to diagnose ambulatory hypertension in clinical practice.

Strengths of our study included the use of a standardized methodology for undertaking ABPM and using ABPM references [6] recommended by the AHA guidelines [1, 3]. The use of OBP references to diagnose office hypertension was consistent with that recommended by the AAP guidelines [3]. Our study limitations include retrospective study design and unavailability of hypertension-induced target-organ damage assessment. Though there is paucity of data on the outcome-based studies with the AHA threshold, 24-h ABP 95th percentile has been shown to have a strong association with hypertension-induced target organ damage [33, 34]. The use of OBP measurements from a single visit may potentially influence the estimation of WCH and MH in our analysis. However, OBP measurements should not change our observations on ambulatory hypertension and the relative pattern of interaction among the ambulatory thresholds to diagnose WCH/MH. Despite the fact that we included participants from two different time periods to enhance the statistical power of our analysis, a consistency over the two time periods in regards to evaluating secondary etiologies of hypertension [3, 24], machines used for OBP/ABP measurements, OBP/ABP thresholds for data interpretation, and no difference in age, sex, BMI z-score, overweight-obese distribution should minimize a potential cofounding effect of using data from two different time periods. A relatively larger representation of primary hypertension and those not on a blood pressure medication limits the generalizability of our findings to those with secondary hypertension and on blood pressure medications. Predominant Caucasian ethnicity limits the applicability of our observations to other ethnicities. In view of the tertiary care setting of our study, our results should be generalized to a primary care population with caution.

## Conclusions

Our study addresses the important question of an appropriate diagnosis of ambulatory hypertension on 24-h ABP monitoring, and consequently that of WCH and MH, by the commonly used ABP thresholds in children. Our findings suggest that 24-h ABP threshold has a strong agreement with the AHA threshold to diagnose ambulatory hypertension, WCH and MH. However, notably both 24-h ABP and AHA thresholds diagnose ambulatory hypertension, WCH, and MH differently than the day-night ABP thresholds. Given the importance choosing an appropriate ABP threshold for diagnosing ambulatory hypertension, our findings are relevant for an appropriate interpretation of ambulatory hypertension, WCH and MH by these commonly used ABP thresholds in clinical practice and for research purposes.

## Data Availability

The datasets generated and analyzed during the current study are not publicly available due to patient’s confidentiality but are available from the corresponding author on reasonable request.

## Abbreviations

AAP: American Academy of Pediatrics
ABP: Ambulatory blood pressure
AHA: American Hypertension Association
ABPM: Ambulatory blood pressure monitoring
BMI: Body mass index
CI: Confidence interval
IQR: Interquartile range
MH: Masked hypertension
OBP: Office blood pressure
SD: Standard deviation
WCH: White coat hypertension
